# Immunohistochemical expression of CANT1 and B3GNT3 in invasive ductal carcinoma of the breast: diagnostic and prognostic significance in lymph node metastasis

**DOI:** 10.1186/s13000-025-01745-9

**Published:** 2026-01-16

**Authors:** Dalia Mostafa Thabet, Al Shaimaa Wagdy Kassem Abu Bakr

**Affiliations:** Department of Pathology, Faculty of Medicine, Minia University, Minia, 61511 Egypt

**Keywords:** CANT1, B3GNT3, Invasive ductal carcinoma, Lymph node metastasis, Prognosis

## Abstract

**Background:**

Breast cancer is a leading global health concern, with lymph node metastasis (LNM) being a key prognostic factor affecting patient outcomes. Glycosylation-related enzymes such as calcium-activated nucleotidase 1 (CANT1) and Beta-1,3-N-acetylglucosaminyltransferase 3 (B3GNT3) have been implicated in tumour progression, yet their roles in breast cancer, particularly invasive ductal carcinoma (IDC), are not well defined. This study investigates the immunohistochemical expression and correlation of CANT1 and B3GNT3 in IDC and their potential role in predicting LNM and clinical outcomes.

**Materials and methods:**

Slides from paraffin blocks of 140 IDC cases and 108 corresponding metastatic axillary lymph nodes were stained with CANT1 and B3GNT3 antibodies. Associations between markers’ immunoreactivity and clinicopathological variables were evaluated. Progression-free survival (PFS) was analysed using the Kaplan–Meier method. The prognostic significance of each variable was evaluated using both univariate and multivariate Cox proportional hazards regression analyses.

**Results:**

High CANT1 and B3GNT3 expression was observed in 47.1% and 45.7% of cases, respectively. Both markers were significantly associated with tumour grade, tumour stage, Nottingham prognostic index, lymph node status, lymph node ratio, Her2 status, Ki-67 proliferative index and distant metastasis. A significant positive correlation was found between CANT1 and B3GNT3 expression (p < 0.001). Co-expression of both markers was strongly associated with LNM, along with a significant difference in the expression levels of each marker between primary tumours and corresponding LNM. Univariate analysis showed that tumour grade, stage, ER status and high B3GNT3 expression were all significantly associated with worse PFS. Multivariate Cox regression identified B3GNT3 expression, tumour grade and tumour stage as independent predictors of poor prognosis in IDC. High expression levels of CANT1 and B3GNT3 were associated with reduced PFS across all IDC cases (*p* = 0.035 and *p* = 0.001, respectively).

**Conclusions:**

High CANT1 and B3GNT3 expressions are associated with aggressive clinicopathological features in IDC and predict unfavourable outcomes. These markers may serve as potential prognostic indicators and independent predictors of LNM in IDC patients.

## Introduction

Breast cancer is a major global health challenge. According to GLOBOCAN 2020 estimates, breast cancer accounts for approximately 2.3 million new cases, representing 11.7% of all cancer diagnoses. It is also the 5th leading cause of cancer-related mortality worldwide [1]. While incidence rates are higher in transitioned countries due to factors such as reproductive patterns, lifestyle, and better detection services, mortality rates are lower in these regions due to access to advanced treatments and well-developed healthcare systems [2]. In contrast, transitioning countries experience higher mortality rates due to limited access to early detection and therapies, compounded by insufficient healthcare infrastructure [3].

Given the complexity of breast cancer, studying the underlying molecular pathways of carcinogenesis is mandatory to predict its behaviour and develop targeted therapies. Despite advancements in early detection procedures, a significant proportion of patients still present with lymph node metastasis (LNM), a crucial pathological feature that plays a central role in prognosis and clinical decision-making [4]. Therefore, identifying novel biomarkers, particularly those involved in glycosylation pathways, extracellular matrix (ECM) remodelling and LNM is vital to enhance diagnostic accuracy, refine patient stratification, and improve prognostic evaluation.

The rationale behind focusing on biomarkers involved in glycosylation pathways and extracellular matrix (ECM) remodelling lies in their well-established roles in tumour progression and metastasis. ECM remodelling facilitates tumour invasion and dissemination to lymph nodes [5]. Additionally, glycosylation, a pivotal post-translational modification, entails the attachment of carbohydrate subunits to proteins and lipids, influencing their structural conformation and functional roles. This process is essential for proper protein folding, stability, cellular signalling, and cellular interactions [6]. In malignant transformations, aberrant glycosylation patterns are frequently observed, disrupting normal cellular interactions and promoting tumourigenic behaviours, such as invasion and metastasis. These alterations underscore the molecular basis of tumour progression and highlight potential avenues for therapeutic intervention [7].

Calcium-activated nucleotidase 1 (CANT1) is an enzyme involved in calcium-dependent nucleotidase metabolism, hydrolysing uridine diphosphate to uridine monophosphate. Elevated CANT1 expression has been linked to tumour progression and poor prognosis in various cancers, including lung adenocarcinoma and hepatocellular carcinoma (HCC) [8, 9]. Beta-1,3-N-acetylglucosaminyltransferase 3 (B3GNT3) is a glycosyltransferase located on chromosome 19q13.1 and involved in the synthesis of poly-N-acetyllactosamine chains. These chains are critical for forming the structural backbone of dimeric sialyl Lewis A and influencing L-selectin ligand function. Overexpression of B3GNT3 is associated with progression in different cancers such as lung adenocarcinoma and oesophageal cancer [10, 11].These findings suggest that CANT1 and B3GNT3 may act as potential biomarkers for cancer diagnosis and prognosis.

To the best of our knowledge, the relationship between CANT1 and B3GNT3 in breast cancer has not previously been explored. Therefore, this work aims to study the immunohistochemical expression and correlation of CANT1 and B3GNT3 in invasive ductal carcinoma (IDC) of the breast to assess their potential significance in predicting LNM and clinical outcomes. Additionally, progression-free survival (PFS) was analyzed using Kaplan–Meier curves, and both univariate and multivariate Cox regression analyses were performed to evaluate the independent prognostic value of each variable.

## Materials and methods

### Tissue specimens

This retrospective analysis included 140 archival paraffin-embedded radical mastectomy specimens collected from patients diagnosed with IDC, along with 108 paraffin blocks of LNM-positive cases corresponding to the primary tumours. Samples were randomly selected from the archives of the Pathology Laboratory at Minia University Hospital, covering the period between January 2017 and January 2019. Exclusion criteria included prior neoadjuvant therapy, non-IDC histological types, and core biopsy specimens. Ethical approval for this study was granted by the Institutional Review Board (IRB) under approval code (1494/2025).

### Clinicopathological data

Clinicopathological data were retrieved from the medical records. Histopathological evaluations were performed on sections stained with haematoxylin and eosin. Tumour staging was assessed according to the most recent TNM classification guidelines set by the American Joint Committee on Cancer (AJCC) [12]. Histological grading followed the Nottingham Histologic Score System (The Elston and Ellis modification of Scarff-Bloom-Richardson grading system) [13] and Nottingham Prognostic Index (NPI) was assessed [14].

Tumor-infiltrating lymphocytes (TILs) were evaluated according to the guidelines of the International TILs Working Group 2014, and were categorized as absent, mild (< 10%), moderate (10–40%), or severe (> 40%) based on the percentage of stromal lymphocytic infiltration [15], the presence of ductal carcinoma in situ (DCIS) as well as lymphovascular invasion (LVI) were evaluated [16, 17]. Lymph node ratio (LNR) was calculated by dividing the number of positive lymph nodes by the total number of excised nodes. Based on established cutoffs, LNR was categorized into three groups: low risk (≤ 0.20), intermediate risk (> 0.20–0.65), and high risk (> 0.65) [18]. Molecular subtypes were determined based on the expression results for oestrogen receptor (ER), progesterone receptor (PR), Her2, and Ki-67 [19].

### Immunohistochemical procedure

Briefly, sections were cut to 5 μm thickness, mounted on positively charged slides, de-paraffinized, and rehydrated. Slides were immersed in 3% hydrogen peroxide for 30 min, rinsed in phosphate-buffered saline (PBS) solution and subjected to antigen retrieval using citrate buffer (pH 6.0) in the microwave. After cooling to room temperature, the slides were washed in PBS. CANT1 mouse monoclonal antibody [concentrated form (100 µl) with a dilution of 1:150, Thermo Fisher Scientific, USA, Catalog No # MA5-26752] and B3GNT3 rabbit polyclonal antibody [concentrated form (100 µl) with a dilution of 1:100, ABClonal, USA, Catalog No # A17611] were applied, followed by overnight incubation at 4 °C in a humidity chamber.

After rinsing with PBS, sections were treated with goat anti-mouse IgG (H + L) secondary antibody [Thermo Fisher Scientific, USA; Cat. No. 31430] for CANT1, and goat anti-rabbit IgG (H + L) secondary antibody [Thermo Fisher Scientific, USA; Cat. No. 31460] for B3GNT3. This was followed by application of the Pierce™ Streptavidin–Biotin Peroxidase Complex (ABC) Kit [Thermo Fisher Scientific, USA; Cat. No. PI-21121], according to the manufacturer’s instructions. Sections were then stained with 3,3'-diaminobenzidine tetrahydrochloride (DAB), washed in distilled water, and counterstained with Harris haematoxylin. Finally, slides were dehydrated, cleared in xylene, and coverslipped. Positive controls (human liver tissue for CANT1 and rat ovary for B3GNT3) were included in each staining batch. Negative controls were processed without primary antibodies.

### Evaluation of immunostaining

All stained sections were reviewed by two independent pathologists in a blinded fashion using an Olympus light microscope (Japan). Cytoplasmic staining of CANT1 and B3GNT3 was considered as positive expression. Immunoreactivity was evaluated using a semiquantitative integration method, calculated by multiplying the staining intensity by the percentage of positive tumour cells for both markers [9, 20].

#### CANT1 expression scoring

For CANT1, immunostaining was scored based on staining intensity and the percentage of positive cells. Staining intensity was evaluated as follows: 0 indicated no staining (colourless), 1 indicated light yellow granules in the cytoplasm, 2 indicated brown granules in the cytoplasm, and 3 indicated strongly brown granules in the cytoplasm. The percentage of positive cells was scored as follows: 0 points for negative, 1 point for < 25%, 2 points for 26–50%, 3 points for 51–75%, and 4 points for > 75%. The degree of positive staining was defined as follows: a score of 1–3 was considered weakly positive (+), 4–6 was moderately positive (+ +), and 7–12 was strongly positive (+ + +). Based on the median value, the results were further divided into low and high expression groups [21].

#### B3GNT3 expression scoring

For B3GNT3, immunostaining was scored based on: (i) staining intensity: 0 (no staining), 1 (weak = light yellow), 2 (moderate = yellow–brown), and 3 (strong = brown); (ii) the percentage of positive tumour cells: 0 (0%), 1 (1%−10%), 2 (11%−50%), 3 (51%−75%), and 4 (76%−100%). The staining index, ranging from 0 to 12, was calculated, with a score ≥ 6 considered high expression and < 6 as low expression [22].

##### Survival data analysis

PFS data were collected from pathology laboratory records at Minia University Hospital, covering cases from January 2019 to December 2023. PFS was defined as the time from diagnosis to disease progression or last follow-up, ranging from 10 to 60 months. The mean ± standard deviation (SD) was 43.8 ± 15.2 months, with a median PFS of 50 months. Kaplan–Meier survival curves were generated for PFS, and the log-rank test was used to compare survival distributions across groups. Mean survival times and 95% confidence intervals were reported [23]. Univariate and multivariate Cox regression analyses were performed to evaluate the prognostic significance of clinicopathological and molecular variables.

##### Statistical analysis

Statistical analysis was performed using the Statistical Package of Social Science (SPSS) version 20 (IBM Corp, Armonk, NY, USA). The association between CANT1 and B3GNT3 expression and the clinicopathological parameters was determined using the Chi-square and Fisher’s exact tests. The correlation between the two markers was evaluated using Chi-square test. The prognostic impact of CANT1 and B3GNT3 expression in IDC was initially assessed using univariate analysis, followed by multivariate Cox regression to evaluate their independent prognostic significance. A p-value of < 0.05 was considered statistically significant for all analyses [24].

## Results

### Clinicopathological data

The study included 140 cases of IDC. The mean age ± SD of the studied cases was 49.47 ± 9.51 and the median age was 50 years (ranged from 25 −75 years). Patients were classified into two groups based on the median age, ≤ 50 years and > 50 years. The mean tumour size ± SD was 3.94 ± 1.9 cm and the median was 4 cm (ranged from 1 to 10 cm). Grade I cases were excluded from the study due to their unavailability. Other Clinicopathological characteristics were summarised in Table 1.

**Table 1:** The clinicopathological data of IDC patients (*n* = 140)

Clinicopathological data		No	Percent%
Age (years)	≤ 50 years	86	61.4%
	> 50 years	54	38.6%
Laterality	Right breast	62	44.3%
	Left breast	78	55.7%
Size (cm)	≤ 2cm	32	22.9%
	> 2cm- ≤ 5cm	72	51.4%
	> 5cm	36	25.7%
Grade	II	102	72.9%
	III	38	27.1%
Lymph node status	0	32	22.9%
	1–3	46	32.8%
	> 3	62	44.3%
LNR	Low risk	38	27.2%
	Intermediate risk	52	37.1%
	High risk	50	35.7%
Tumour stage	I	16	11.4%
	II	34	24.3%
	III	68	48.6%
	IV	22	15.7%
NPI	Good	16	11.4%
	Moderate	52	37.1%
	Poor	72	51.5%
ER	Positive	78	55.7%
	Negative	62	44.3%
PR	Positive	70	50%
	Negative	70	50%
HER2- enriched	Positive	44	31.4%
	Negative	96	68.6%
Ki-67 proliferative index	≤ 30%	66	47.1%
	> 30%	74	52.9%
Molecular types	Luminal A	46	32.9%
	Luminal B	52	37.1%
	HER subtype	22	15.7%
	Triple negative	20	14.3%
Tumour necrosis	Present	20	14.3%
	Absent	120	85.7%
Local recurrence	Present	18	12.9%
	Absent	122	87.1%
TILs	Absent	12	8.6%
	Mild	34	24.3%
	Moderate	40	28.6%
	Severe	54	38.5%
DCIS	Present	80	57.1%
	Absent	60	42.9%
LVI	Present	60	42.9%
	Absent	80	57.1%
Distant metastasis	Present	22	15.7%
	Absent	118	84.3%

HER2-enriched” refers to HER2 positivity based on immunohistochemistry (IHC), regardless of hormone receptor status. The “HER2 subtype,” as defined by molecular classification, includes only tumors that are HER2-positive and hormone receptor-negative (ER −/PR −)

*NPI* Nottingham prognostic index, *LVI* lymphovascular invasion, *LNR* Lymph node ratio, *DCIS*: Ductal carcinoma in situ, *TILs* Tumour infiltrating lymphocytes

### Immunohistochemical expression of CANT1 and its association with clinicopathological data

**Fig. 1:**
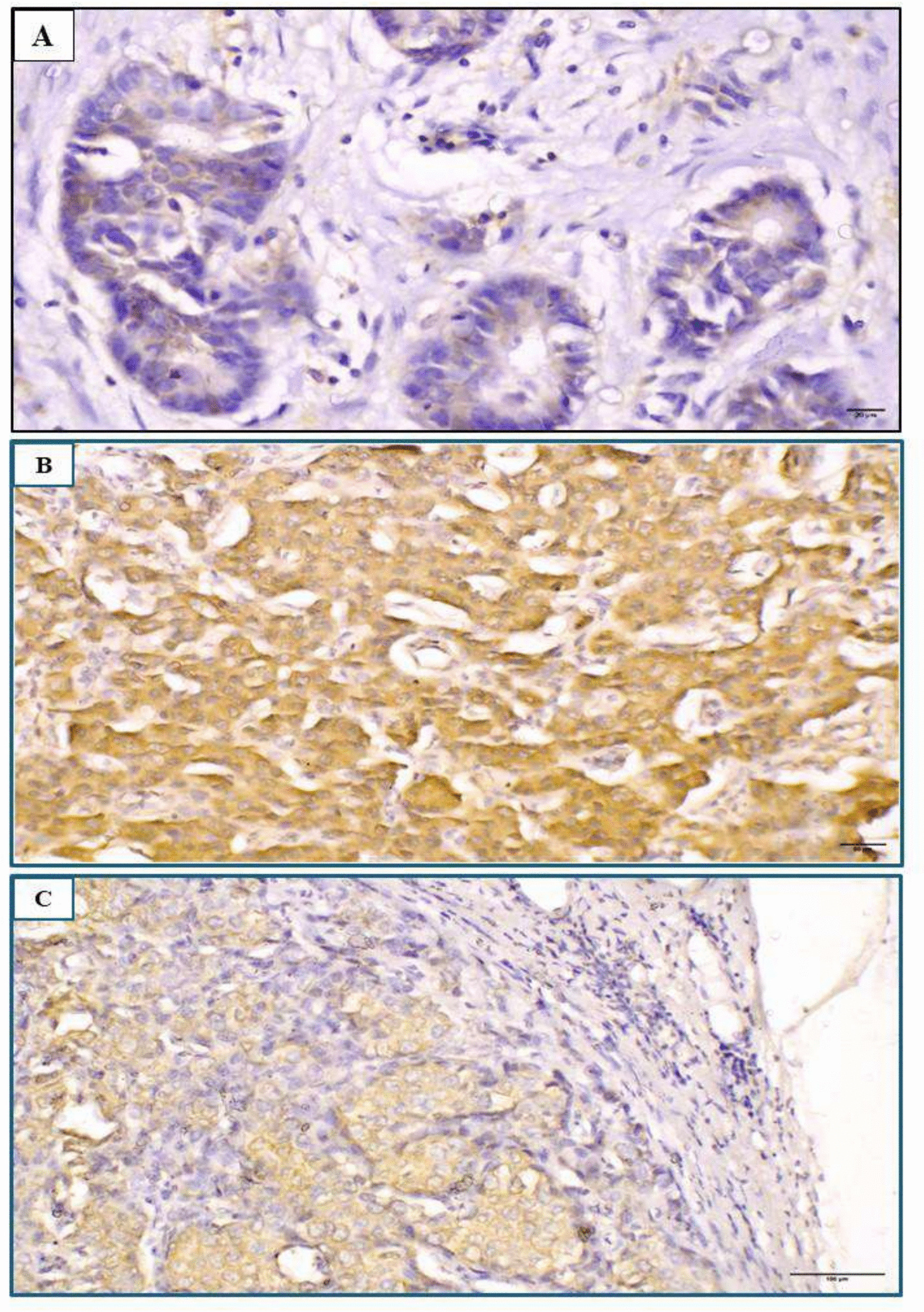
Representative photomicrographs showing immunohistochemical expression of CANT1 in invasive ductal carcinoma of the breast (primary tumor and metastatic lymph node). **A** Low cytoplasmic expression in a grade II tumour (original magnification X400; scale bar = 20 μm). **B** High cytoplasmic expression in a grade III tumour (original magnification × 200; scale bar = 50 μm). **C** High expression in a corresponding metastatic lymph node (original magnification × 100; scale bar = 100 μm)

In the present study, 47.1% (66/140 cases) revealed high CANT1 expression, while 52.9% (74/140 cases) showed low cytoplasmic expression (Fig. 1). A significant association was detected between high CANT1 expression and several clinicopathological factors, including tumour size (*p* = 0.004), grade (*p* = 0.029), stage (*p* = 0.001), LNR (*p* = 0.026), lymph node status (*p* = 0.028), NPI (*p* = 0.008), PR status (*p* = 0.031), Her2 status (*p* = 0.024), Ki-67 proliferative index (*p* = 0.029) and distant metastasis (*p* = 0.002) as detailed in Table 3. No significant associations were found between CANT1 expression and the remaining clinicopathological parameters.

**Table 2:** Association between cytoplasmic CANT1 expression and clinicopathological data for the patients with IDC (*n* = 140)

Clinicopathological data	No	CANT1 cytoplasmic expression		*P* value
		**Low expression (%) 74 (52.9%)**	**High expression (%) 66 (47.1%)**	
Age (years)				
≤ 50	86	40 (46.5)	46 (53.5)	0.180
> 50	54	34 (63)	20 (37)	
Laterality				
Right breast	62	34 (54.8)	28 (45.2)	0.767
Left breast	78	40 (51.3)	38 (48.7)	
Size (cm)				
< 2 cm	32	28 (87.5)	4 (12.5)	0.004*
> 2—≤ 5 cm	72	34 (47.2)	38 (52.8)	
> 5 cm	36	12 (33.3)	24 (66.7)	
Grade				
II	102	62 (60.8)	40 (39.2)	0.029*
III	38	12 (31.6)	26 (68.4)	
Lymph node status				
0	32	26 (81.2)	6 (18.8)	0.028*
1–3	46	18 (39.1)	28 (60.9)	
> 3	62	30 (48.4)	32 (51.6)	
LNR				
Low risk	38	34 (89.5)	4 (10.5)	0.026*
Intermediate risk	52	24 (46.2)	28 (53.8)	
High risk	50	16 (32)	34 (68)	
Tumour stage				
I	16	16 (100)	0 (0)	0.001*
II	34	20 (58.8)	14 (41.2)	
III	68	36 (52.9)	32 (47.1)	
IV	22	2 (9.1)	20 (90.9)	
NPI				
Good	16	16 (100)	0 (0)	0.008*
Moderate	52	28 (53.8)	24 (46.2)	
Poor	72	30 (41.7)	42 (58.3)	
ER				
Positive	78	44 (56.4)	34 (43.6)	0.504
Negative	62	30 (48.4)	32 (51.6)	
PR				
Positive	70	46 (65.7)	24 (34.3)	0.031*
Negative	70	28 (40)	42 (60)	
HER2- enriched				
Positive	44	32 (72.7)	12 (27.3)	0.024*
Negative	96	42 (43.8)	54 (56.2)	
Ki-67 proliferative index				
≤ 30%	66	44 (66.7)	22 (33.3)	0.029*
> 30%	74	30 (40.5)	44 (59.5)	
Molecular subtypes				
Luminal A	46	32 (69.6)	14 (30.4)	0.267
Luminal B	52	24 (46.2)	28 (53.8)	
HER2 subtype	22	10 (45.5)	12 (54.5)	
Triple negative	20	8 (40)	12 (60)	
Tumour necrosis				
Present	20	12 (60)	8 (40)	0.739
Absent	120	62 (51.7)	58 (48.3)	
Local Recurrence				
Present	18	8 (44.4)	10 (55.6)	0.726
Absent	122	66 (54.1)	56 (45.9)	
TILs				
Absent	12	6 (50)	6 (50)	0.616
Mild	34	22 (64.7)	12 (35.3)	
Moderate	40	22 (55)	18 (45)	
Severe	54	24 (44.4)	30 (55.6)	
DCIS				
Present	80	48 (60)	32 (40)	0.167
Absent	60	26 (43.3)	34 (56.7)	
LVI				
Present	60	30 (50)	30 (50)	0.678
Absent	80	44 (55)	36 (45)	
Distant metastasis				
Present	22	2 (9.1)	20 (90.9)	0.002*
Absent	118	72 (61)	46 (39)	

Test of significance: Chi-Square and Fisher's exact tests

HER2-enriched” refers to HER2 positivity based on immunohistochemistry (IHC), regardless of hormone receptor status. The “HER2 subtype,” as defined by molecular classification, includes only tumors that are HER2-positive and hormone receptor-negative (ER −/PR −)

*NPI* Nottingham prognostic index, *LVI* Lymphovascular invasion, *LNR* Lymph node ratio, *DCIS* Ductal carcinoma in situ, *TILs* Tumour infiltrating lymphocytes

^*^P—value < 0.05 is considered statistically significant

### Immunohistochemical expression of B3GNT3 and its association with clinicopathological data

**Fig. 2:**
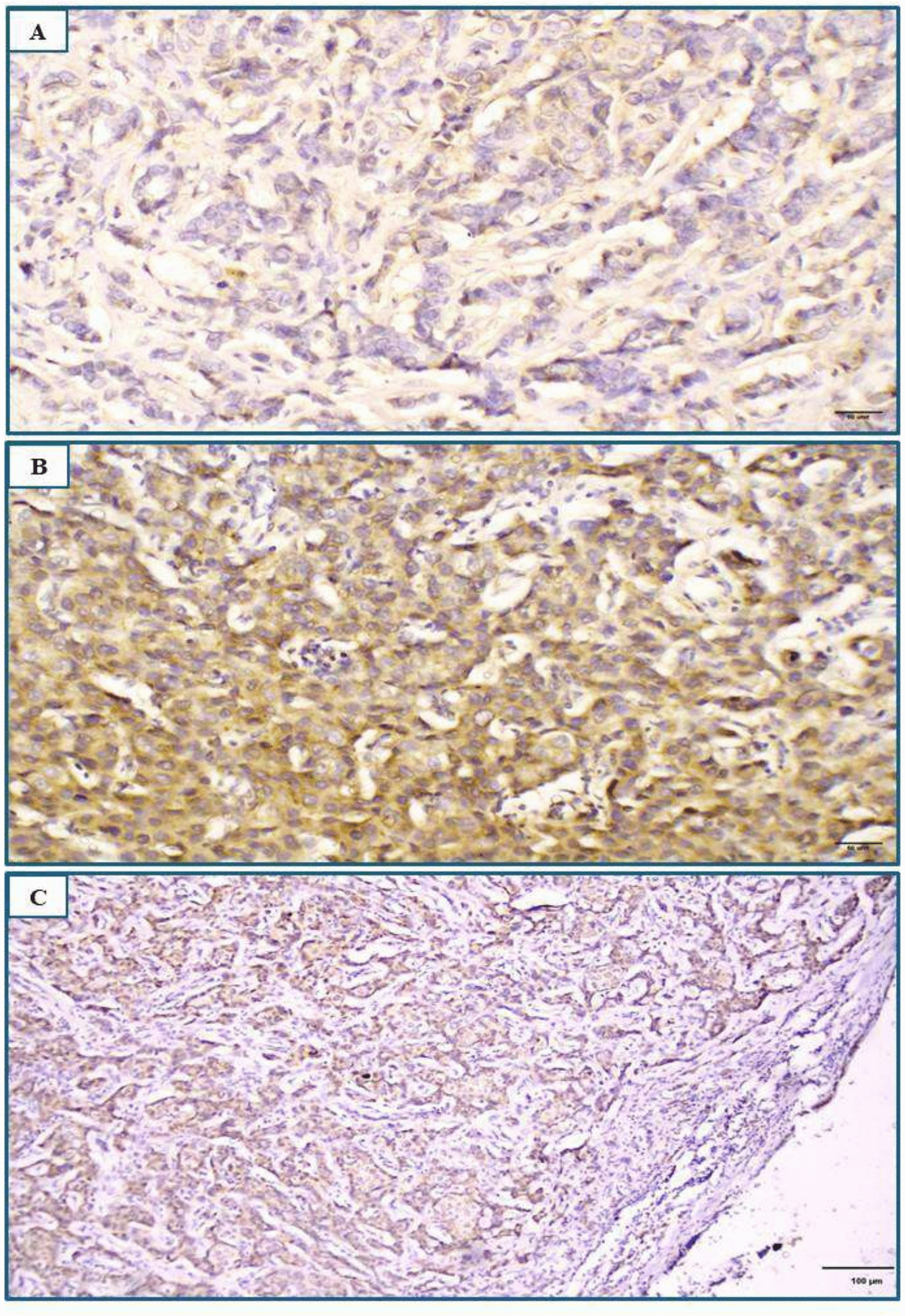
Representative photomicrographs of immunohistochemical expression of B3GNT3 in invasive ductal carcinoma of the breast (primary tumor and metastatic lymph node). **A** Low cytoplasmic expression in a grade II tumour (original magnification × 200; scale bar = 50 μm). **B** High cytoplasmic expression in a grade III tumour (original magnification × 200; scale bar = 50 μm). **C** High expression in a corresponding metastatic lymph node (original magnification × 100; scale bar = 100 μm)

In the current study, 45.7% (64/140 cases) revealed high cytoplasmic B3GNT3 expression, while 54.3% (76/140 cases) revealed low expression (Fig. 2). A significant association was detected between high B3GNT3 expression and several clinicopathological factors, including tumour grade (*p* = 0.020), stage (*p* < 0.001), lymph node status (*p* = 0.001), LNR (*p* = 0.005), NPI (*p* = 0.001), Her2 status (*p* = 0.002), Ki-67 proliferative index (p = 0.003) and distant metastasis (*p* < 0.001) as shown in Table 3. No significant associations were found between B3GNT3 expression and the other clinicopathological data.

**Table 3:** Association between B3GNT3 cytoplasmic expression and clinicopathological data for the patients with IDC (*n* = 140)

Clinicopathological data	No	B3GNT3 cytoplasmic expression			*P* value
		**Low expression (%) 76 (54.3%)**	**High expression (%) 64 (45.7%)**		
Age (years)					
≤ 50	86	50 (58.1)	36 (41.9)		0.414
> 50	54	26 (48.1)	28 (51.9)		
Laterality					
Right breast	62	28 (45.2)	34 (54.8)		0.172
Left breast	78	48 (61.5)	30 (38.5)		
Size (cm)					
< 2 cm	32	24 (75)	8 (25)		0.163
> 2—≤ 5 cm	72	34 (47.2)	38 (52.8)		
> 5 cm	36	18 (50)	18 (50)		
Grade					
II	102	64 (62.7)	38 (37.3)		0.020*
III	38	12 (31.6)	26 (68.4)		
Lymph node status					
0	32	30 (93.8)	2 (6.2)		0.001*
1–3	46	20 (43.5)	26 (56.5)		
> 3	62	26 (41.9)	36 (58.1)		
LNR					
Low risk	38	32 (84.2)	6 (15.8)		0.005*
Intermediate risk	52	26 (50)	26 (50)		
High risk	50	18 (36)	32 (64)		
Tumour stage					
I	16	14 (87.5)		2 (12.5)	< 0.001*
II	34	26 (76.5)		8 (23.5)	
III	68	36 (52.9)		32 (47.1)	
IV	22	0 (0)		22 (100)	
NPI					
Good	16	14 (87.5)		2 (12.5)	0.001*
Moderate	52	38 (73.1)		14 (26.9)	
Poor	72	24 (33.3)		48 (66.7)	
ER					
Positive	78	50 (64.1)		28 (35.9)	0.064
Negative	62	26 (41.9)		36 (58.1)	
PR					
Positive	70	46 (65.7)		24 (34.3)	0.055
Negative	70	30 (42.9)		40 (57.1)	
HER2- enriched					
Positive	44	36 (81.8)		8 (18.2)	0.002*
Negative	96	40 (41.7)		56 (58.3)	
Ki-67 proliferative index					
≤ 30%	66	48 (60.6)		18 (39.4)	0.003*
> 30%	74	28 (48.6)		46 (51.4)	
Molecular subtypes					
Luminal A	46	30 (65.2)		16 (34.8)	0.324
Luminal B	52	30 (57.7)		22 (42.3)	
HER2 subtype	22	8 (36.4)		14 (63.6)	
Triple negative	20	8 (40)		12 (60)	
Tumour necrosis					
Present	20	14 (70)		6 (30)	0.326
Absent	120	62 (51.7)		58 (48.3)	
Local Recurrence					
Present	18	8 (44.4)		10 (55.6)	0.722
Absent	122	68 (55.7)		54 (44.3)	
TILs					
Absent	12	6 (50)		6 (50)	0.11
Mild	34	24 (70.6)		10 (29.4)	
Moderate	40	26 (65)		14 (35)	
Severe	54	20 (37)		34 (63)	
DCIS					
Present	80	46 (57.5)		34 (42.5)	0.167
Absent	60	30 (50)		30 (50)	
LVI					
Present	60	34 (56.7)		26 (43.3)	0.75
Absent	80	42 (52.5)		38 (47.5)	
Distant metastasis					
Present	22	0 (0)		22 (100)	< 0.001*
Absent	118	76 (64.4)		42 (35.6)	

Test of significance: Chi-Square and Fisher's exact tests

HER2-enriched” refers to HER2 positivity based on immunohistochemistry (IHC), regardless of hormone receptor status. The “HER2 subtype,” as defined by molecular classification, includes only tumors that are HER2-positive and hormone receptor-negative (ER −/PR −)

*NPI* Nottingham prognostic index, *LVI* Lymphovascular invasion, *LNR* Lymph node ratio, *DCIS* Ductal carcinoma in situ, *TILs* Tumour infiltrating lymphocytes

^*^P—value < 0.05 is considered statistically significant

### Correlation between immunohistochemical expression of examined markers

**Fig. 3:**
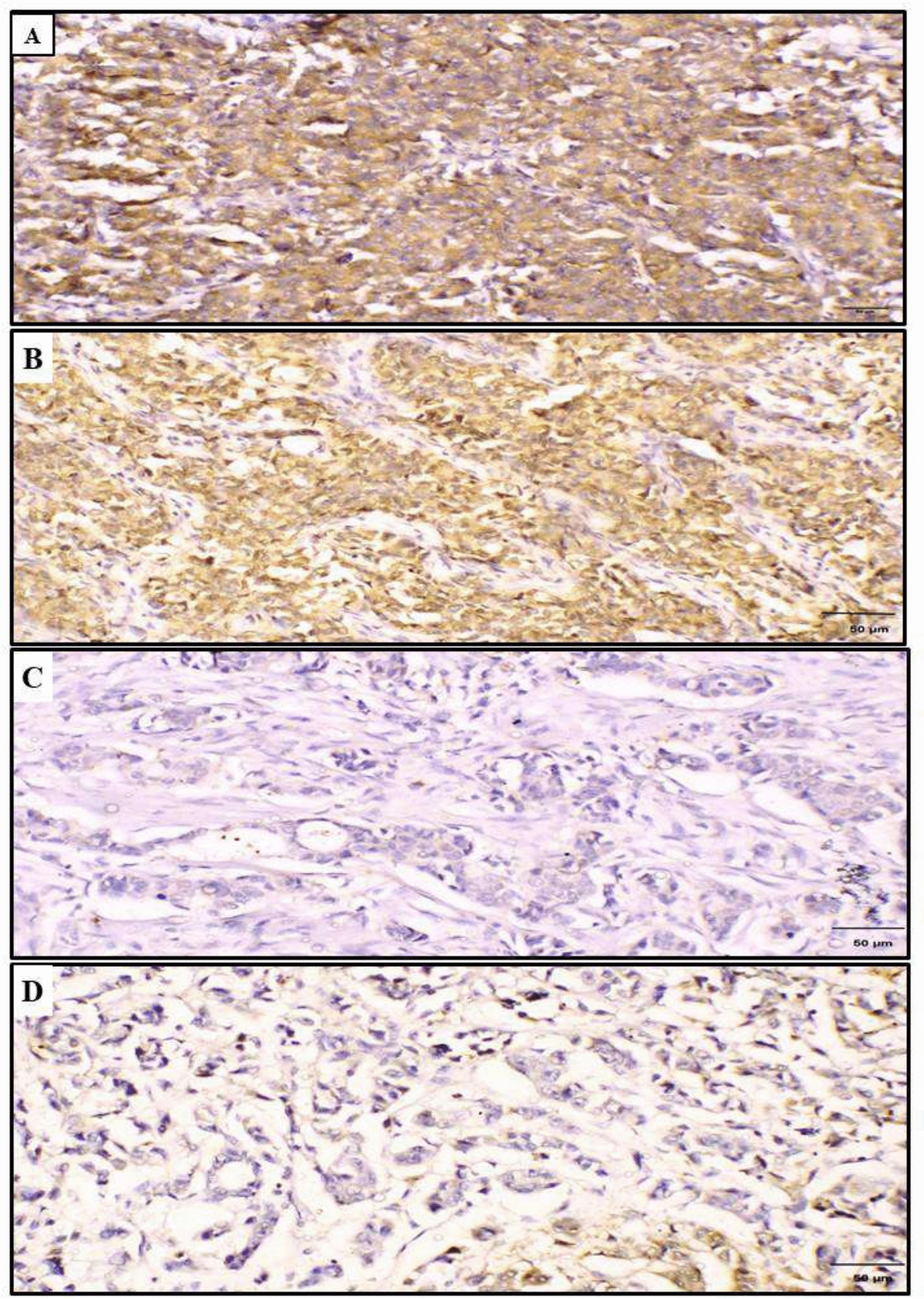
Representative photomicrographs of combined immunohistochemical expression of CANT1 and B3GNT3 in invasive ductal carcinoma of the breast. **A** High CANT1 expression. **B** High B3GNT3 expression. **C** Low CANT1 expression. **D** Low B3GNT3 expression (All images at original magnification × 200; scale bar = 50 μm)

There was a statistically significant positive correlation between CANT1 expression and B3GNT3 (*p* < *0.001, r* = *0.627)* as shown in Fig. 3 and Table 4.

**Table 4:** Correlation between immunohistochemical expression of CANT1 and B3GNT3 (*n* = 140)

		**CANT1**		**Total**	***P* value**
		**Low (%)**	**High (%)**		
B3GNT3	Low	62 (83.8)	12 (16.2)	74	< 0.001*
	High	14 (21.2)	52 (78.8)	66	
Total		76 (54.3)	64 (45.7)	140	

Test of significance: Chi-square test

^*^P—value < 0.05 is considered statistically significant

### Survival analysis and prognostic significance

**Fig. 4:**
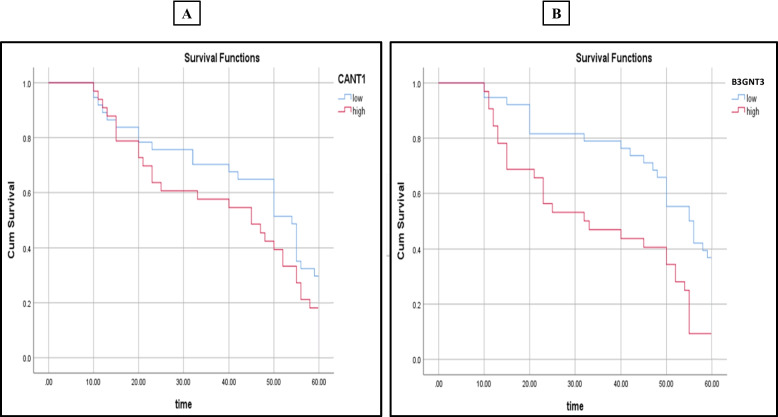
Kaplan–Meier survival curves illustrating progression-free survival (PFS) for all IDC cases (*n* = 140), stratified by immunoexpression levels of CANT1 (**A**) and B3GNT3 (**B**). High expression of both markers is significantly associated with reduced PFS (*p* < 0.05, log-rank test)

We investigated the PFS of 140 cases of IDC patients, analysing variables including marker expression and clinicopathological characteristics. The PFS ranged from 10 to 60 months with a mean ± SD of 43.8 ± 15.2 months and the median survival time was 50 months. Patients with high CANT1 and B3GNT3 immunoexpression had significantly shorter PFS than those patients with low expression (*p* = 0.035 and *p* = 0.001, respectively) as shown in Fig. 4.

Univariate analysis was conducted to identify factors significantly associated with PFS. Several clinicopathological and molecular variables demonstrated statistically significant associations with PFS. They included tumour grade (*p* = 0.006), stage (*p* < 0.001), and ER status (*p* = 0.003). Additionally, high B3GNT3 expression was significantly correlated with worse PFS (*p* = 0.005). (Table 5).

**Table 5:** Univariate Analysis of Progression-Free Survival (PFS) in IDC Patients (*n* = 140)

		**Mean PFS (months)**	**HR**	**95% CI**		***P*-value**
				**Lower bound**	**Upper bound**	
Age	≤ 50 years	43.34	1.0	-	-	0.25
	> 50 years	38.63	1.33	0.82	2.16	
Laterality	Right	41.61	1.0	-	-	0.64
	Left	41.51	0.89	0.56	1.43	
Tumour size	≤ 2cm	44.38	1.0	-	-	0.30
	2-5cm	39.64	1.30	0.85	1.98	
	> 5 cm	42.89	1.08	0.81	2.10	
Tumour grade	Grade II	45.08	1.0	-	-	0.006*
	Grade III	32.11	2.15	1.25	3.71	
DCIS	Absent	42.40	1.0	-	-	0.23
	Present	40.43	1.34	0.83	2.17	
Tumour necrosis	Absent	46.70	1.0	-	-	0.66
	Present	40.70	1.17	0.60	2.28	
LVI	Absent	50.46	1.0	-	-	0.93
	Present	39.90	1.36	0.71	2.54	
Lymph node status	0	51.25	1.0	-	-	0.08
	1–3	49.94	1.23	0.90	1.68	
	> 3	41.05	1.45	0.98	2.15	
LNR	Low risk	47.95	1.0	-	-	0.29
	Intermediate risk	38.58	1.24	0.85	1.80	
	High risk	39.80	1.20	0.82	1.75	
NPI	Good	44.75	1.0	-	-	0.50
	Moderate	45.00	0.96	0.75	1.25	
	Poor	38.36	1.27	0.86	1.89	
Stage	Stage I	45.43	1.0	-	-	< 0.001*
	Stage II	48.12	0.91	0.70	1.20	
	Stage III	44.40	1.20	1.07	1.50	
	Stage IV	19.91	2.71	2.15	5.56	
TILs	Absent	51.25	1.0	-	-	0.31
	Mild	49.94	1.22	0.85	1.75	
	Moderate	41.05	1.45	0.94	2.21	
	Severe	36.13	1.62	0.98	2.68	
ER status	Positive	46.82	1.0	-	-	0.003*
	Negative	34.94	4.11	1.62	10.39	
PR status	Positive	45.20	1.0	-	-	0.46
	Negative	37.91	1.34	0.67	2.90	
HER2-enriched	Positive	44.87	1.0	-	-	0.15
	Negative	40.04	1.46	0.88	2.44	
Ki-67 proliferative index	≤ 30%	43.94	1.0	-	-	0.41
	> 30%	39.43	1.22	0.76	1.95	
Molecular subtypes	Luminal A	39.74	1.0	-	-	0.08
	Luminal B	46.85	0.73	0.52	1.02	
	HER2	40.27	1.12	0.78	1.60	
	Triple negative	33.40	1.68	0.97	2.35	
CANT1	Low	44.16	1.0	-	-	0.26
	High	38.64	1.31	0.82	2.10	
B3GNT3	Low	47.47	1.0	-	-	0.005*
	High	34.53	1.85	1.23	3.26	

*HR* Hazard Ratio, *CI* Confidence Interval, *PFS* Progression-Free Survival

*NPI *Nottingham prognostic index*, LVI *Lymphovascular invasion*, LNR *Lymph node ratio*, DCIS *Ductal carcinoma in situ*, ER *Estrogen Receptor*, PR *Progesterone Receptor, *TILs* Tumour infiltrating lymphocytes

^*^Mean estimates (months) are derived from Kaplan–Meier analysis. Hazard Ratios, 95% CIs, and *P*-values are derived from univariate Cox proportional hazards regression

^*^Reference category for each variable is indicated by HR = 1.0

^**^P-value < 0.05 was considered statistically significant

In multivariate Cox regression analysis, tumour grade (*p* = 0.021), tumour stage (*p* = 0.038) and B3GNT3 expression (*p* = 0.026) were identified as independent prognostic factors for PFS. CANT1 was excluded from the multivariate analysis due to its lack of significance in univariate analysis (*p* = 0.26), while ER status was not statistically significant (Table 6).

**Table 6:** Multivariate Cox regression analysis of progression-free survival (PFS) predictors in IDC patients

	**Sig**	**HR**	**95%CI**	
			**Lower**	**Upper**
B3GNT3 expression	0.026*	1.75	1.07	2.87
Tumour stage	0.038*	1.43	1.02	2.01
Tumour grade	0.021*	1.96	1.11	3.48
ER status	0.35	1.28	0.76	2.16

Hazard ratios (HR), 95% confidence intervals (CI)

Test of significance: COX proportional hazards regression

All variables with *p* < 0.05 were included

^*^P—value < 0.05 is considered statistically significant

### Comparison between the expression of CANT1 in the primary tumour and corresponding LNM

In the analysis of CANT1 expression in 108 paired samples of primary tumours and their corresponding metastatic axillary lymph nodes, 94 pairs (87%) showed a concordance between the primary tumour and metastatic lymph node CANT1 expression. In contrast, 14 cases (13%) exhibited a discordant CANT1 expression between the primary tumour and its corresponding LNM. Specifically, 8 cases (7.4%) showed high expression in the primary tumour, but low expression in the corresponding lymph nodes. Six cases (5.6%) showed low expression in the primary tumour, but high expression in the corresponding LNM. This discordance was statistically significant (*p* < 0.001), indicating that while most cases maintain CANT1 expression status between the primary site and LNM, a notable minority do not, as shown in Table 7.

**Table 7:** Comparison of CANT1 expression between primary tumour and corresponding metastatic lymph nodes (*n* = 108)

CANT1 expression in primary tumour and LNM				*P* value
Concordant expression (*n* = 94)		Discordant expression (*n* = 14)		< 0.001*
High in both primary tumour and LNM (%)	Low in both primary tumour and LNM (%)	Low in primary tumour, high in LNM (%)	High in primary tumour, low in LNM (%)	
52 (48.1)	42 (38.9)	6 (5.6)	8 (7.4)	

Test of significance: Chi-Square test

^*^P—value < 0.05 is considered statistically significant

An additional analysis of hormone receptor status (ER, PR, HER2) in these 14 discordant CANT1 cases revealed that receptor profiles were concordant between the primary tumour and LNM in the vast majority of cases (12/14, 85.7%).

### Comparison between the expression of B3GNT3 in the primary tumour and corresponding LNM

Considering B3GNT3 expression in the primary tumour and corresponding metastatic lymph node in 108 pairs; 102 pairs (94.4%) revealed a concordance expression between the primary tumour and corresponding LNM. However, 6 cases (5.6%) showed different expression of B3GNT3 between primary tumour and corresponding LNM. Two cases (1.8%) showed high expression in primary tumour and low expression in corresponding lymph node, while 4 cases (3.7%) showed low expression in primary tumour and high expression in corresponding metastatic lymph node. The results were statistically significant *(p* < *0.001)* (Table 8).

**Table 8:** Comparison between the expression of B3GNT3 in 108 pairs of primary tumour and corresponding metastatic lymph nodes (*n* = 108)

B3GNT3 expression in primary tumour and LNM				*P* value
Concordant expression (*n* = 102)		Discordant expression (*n* = 6)		< 0.001*
High in primary tumour and LNM (%)	Low in primary tumour and LNM (%)	Low in primary tumour, high in LNM (%)	High in primary tumour, low in LNM (%)	
60 (55.6)	42 (38.9)	4 (3.7)	2 (1.8)	

Test of significance: Chi-Square test

^*^P—value < 0.05 is considered statistically significant

Notably, in all 6 cases with discordant B3GNT3 expression, the receptor status (ER, PR, HER2) remained entirely concordant (100%) between the primary tumour and metastatic lymph nodes.

### Correlation between immunohistochemical co-expression of CANT1 and B3GNT3 with lymph node status

There was a statistically significant positive correlation between the co-expression of CANT1 and B3GNT3 and lymph node involvement (*p* < 0.001, r = 0.559), as presented in Table 9.

**Table 9:** Correlation between immunohistochemical co-expression of CANT1 and B3GNT3 with lymph node status (*n* = 140)

Lymph node status	CANT1 and B3GNT3 co-expression				Total	*P* value
	**Both high (%)**	**Both low (%)**	**High CANT1, low B3GNT3 (%)**	**Low CANT1, high B3GNT3 (%)**		
Negative for LNM	3 (9.4)	26 (81.3)	2 (6.2)	1 (3.1)	32 (22.9)	< 0.001*
Positive for LNM	49 (45.4)	36 (33.3)	10 (9.3)	13 (12)	108 (77.1)	
Total	52 (37.1)	62 (44.3)	12 (8.6)	14 (10)	140	

Test of significance: Chi-Square test

^*^P—value < 0.05 is considered statistically significant

## Discussion

Breast cancer is the second-leading cause of cancer- related mortality among Egyptian women. Compared to developed countries, it is often diagnosed at a younger age in Egypt, with a substantial proportion of patients presenting before the age of 50. This earlier onset is frequently associated with aggressive characteristics, including higher tumour grades and increased incidences of LNM [25]. Consequently, identifying reliable biomarkers for early detection and prognosis is crucial for improving patient outcomes and guiding therapeutic decisions.

CANT1 is an enzyme involved in nucleotide metabolism and glycosylation processes, which play vital roles in cellular functions such as adhesion, migration, and invasion. Although the exact role of CANT1 in breast cancer remains underexplored, studies in other tissues suggest that it may influence tumour progression through its involvement in protein glycosylation. This process is essential for cancer cell dissemination and metastasis to distant organs, including lymph nodes, by altering cell–cell interactions and promoting epithelial-mesenchymal transition (EMT) [26].

B3GNT3, a glycosyltransferase responsible for synthesizing core-1 and core-3 O-glycans, also plays a pivotal role in the biosynthesis of selectin ligands that mediate cell–cell interactions [27]. While B3GNT3's role in other cancers is documented, its association with breast cancer has not been previously well investigated. This study provides novel insights into the relationship between CANT1 and B3GNT3 expression in IDC, contributing to the understanding of their roles in metastasis and disease progression.

In the current study, 47.1% of the cases revealed high cytoplasmic CANT1 expression, while 52.9% of the cases revealed low expression. We found a significant association between high CANT1 expression and larger tumour size (p = 0.004). In terms of tumour grade, high CANT1 expression was found in 39.2% and 68.4% of grade II and III cases, respectively, displaying a significant association with advanced tumour grade. Also, 90.9% of stage IV cases showed high expression, meanwhile all stage I cases (100%) showed low CANT1 expression, and the association was statistically significant (p = 0.001).

These findings could be attributed to the anti-apoptotic role of CANT1. It was discovered that CANT1 knocking down, caused upregulation of the pro-apoptotic factors of BAD and cleaved caspase-3, while the anti-apoptotic factor BCL2 was downregulated [28]. Additionally, CANT1 overexpression had a significant effect on tumourigenesis through promoting of the cell cycle at S phase, which caused progression of the mitotic activity of proliferating cells [29].

Our findings are in accordance with. Liu et al., who detected a significant association between CANT1 expression and larger tumour size, advanced stage, and higher histological grade in hepatocellular carcinoma (HCC) [21]. Similarly, Yao et al., reported a significant association between CANT1 expression with advanced tumour stage in lung adenocarcinoma [9].

In addition, our findings demonstrate that CANT1 expression significantly correlates with LNR, lymph node status, NPI, PR status, HER2 status, Ki-67 proliferative index and distant metastasis. Recent evidence suggests that CANT1 may promote tumour metastasis through activation of the PI3K/AKT pathway, a critical signalling cascade that regulates cell proliferation and survival [30]. By enhancing this pathway, CANT1 can facilitate EMT, a process that enables cancer cells to acquire invasive properties and disseminate to distant organs [26]. These findings align with a previous study that reported a significant association between high CANT1 expression and nodal status and poor survival in lung adenocarcinoma [28]. To our knowledge, no prior studies have explored the link between CANT1 expression and ER status, Her2 status, Ki-67 proliferative index, NPI or LNR in IDC.

Similarly, high cytoplasmic B3GNT3 expression was revealed in 45.7% of the cases, while 54.3% of the cases revealed low expression. B3GNT3 expression was significantly associated with tumour grade, stage, LNR, lymph node status, NPI, Her2 status, Ki-67 proliferative index and distant metastasis. These findings were consistent with previous studies which reported that B3GNT3 high expression was linked to advanced tumour stage, higher grade and poor survival in pancreatic adenocarcinoma, early-stage cervical cancer and lung adenocarcinoma, respectively [20, 22, 31].

The mechanism by which B3GNT3 contributes to carcinogenesis differs across various tumour types, suggesting multiple tumour-specific pathways. Zhuang et al. have explained that B3GNT3 is highly expressed in pancreatic cancer stem cells [32]. Wang et al. indicated that B3GNT3 may promote the growth and migration of endometrial cancer cells by regulating the RhoA/RAC1 signalling pathway [33]. In non-Hodgkin’s lymphoma, B3GNT3 plays dominant roles in L-selectin ligand biosynthesis, which is important for tumour cell survival [22]. In triple-negative breast cancer, B3GNT3 is overexpressed and facilitates PD-L1 glycosylation, thereby enhancing immune checkpoint therapy efficacy [34].

To the best of our knowledge, this is the first study to investigate the relationship between CANT1 and B3GNT3 in IDC. A significant positive correlation was observed between their expression levels (p < 0.001, r = 0.627), suggesting a potential synergistic effect in promoting cancer cell dissemination through shared molecular pathways involved in glycosylation, cell adhesion and ECM interaction. CANT1 supplies nucleotide sugars for glycosylation, while B3GNT3 synthesizes poly-N-acetyllactosamine structures, contributing to altered cell adhesion and immune interactions [35, 36]. In IDC, this interaction may promote tumour cell invasion and enhance metastatic potential.

Interestingly, our results showed that high expression levels of CANT1 and B3GNT3 were significantly associated with negative HER2 status, despite being linked to higher tumour grade and more aggressive features. This inverse association is somewhat paradoxical because HER2 positivity is often related to more aggressive breast cancers. However, HER2-negative tumours, particularly those classified as triple-negative breast cancer (TNBC), can also exhibit highly aggressive behavior and poor prognosis [37]. This highlights the complexity of glycosylation-related pathways in different oncogenic contexts [7]. Since there are no previous studies examining the relationship between CANT1 and B3GNT3 expression and HER2 status in breast cancer, the exact reason for this contradiction is unclear. It may suggest that these markers contribute to tumour aggressiveness through HER2-independent mechanisms [38], indicating a complex and heterogeneous tumour biology. Further studies are needed to explore these pathways and clarify the clinical significance of this finding.

Univariate analysis identified several factors significantly associated with PFS, including tumour grade, stage, and ER status. Additionally, high B3GNT3 expression was linked to worse PFS. Multivariate Cox regression analysis further corroborated these findings, identifying B3GNT3 expression, tumour grade and tumour stage as independent poor prognostic factors for IDC. Similarly, Wu et al., reported thar B3GNT3 was an independent prognostic factor for poor overall survival in lung adenocarcinoma [31]. Interestingly, high expression of CANT1 and B3GNT3 has been associated with favourable prognostic outcomes in prostate cancer and neuroblastoma, respectively [29, 39].

To our knowledge, this is the first study to investigate the association between CANT1 and B3GNT3 expression and PFS in IDC, using both univariate and multivariate analyses. Our findings revealed that high expression of both CANT1 and B3GNT3 was significantly associated with reduced PFS across all IDC cases.

From a clinical perspective, early prediction of LNM is essential for guiding the extent of surgery and the selection of adjuvant therapy strategies [40]. Our findings suggest that CANT1 and B3GNT3 could serve as predictive biomarkers for lymph node involvement. We detected a strong significant association between the co-expression of both markers and LNM (*p* < 0.001, r = 0.527). Additionally, the significant correlations between high expression of each marker with both LNR and lymph node status further supports their potential utility as combined prognostic indicators. Additionally, the significant differences in the expression levels of each marker between the primary tumour and corresponding metastatic lymph nodes indicate their dynamic role in the metastatic cascade.

Previous studies have highlighted the importance of B3GNT3 in the biosynthesis of selectin ligands, which are crucial for lymphocyte homing and cancer cell trafficking to lymph nodes. Furthermore, overexpression of B3GNT3 has been linked to increased LNM in early-stage cervical cancer [22]. Similarly, CANT1 regulates nucleotide metabolism and glycosylation processes that influence the post-translational modification of cell surface proteins involved in adhesion and migration [36]. CANT1 overexpression may also activate the PI3K/AKT signalling pathway, promoting lymphangiogenesis and facilitating cancer cell survival in lymphatic circulation [30].

The expression levels of CANT1 and B3GNT3 show significant potential as diagnostic biomarkers for LNM in IDC. Current diagnostic approaches, including sentinel lymph node biopsy, have limitations, such as false negatives and unnecessary procedures for low-risk patients [41]. Incorporating CANT1 and B3GNT3 expression analysis into routine pathological workflows could improve the accuracy of nodal involvement detection, enabling more precise stratification of patients. Individuals with high biomarker expression may benefit from more intensive nodal assessment, while low expression cases could potentially avoid unnecessary interventions.

Enhancing LNM diagnosis through biomarker integration could lead to more reliable pathological assessments, complementing traditional histopathological techniques and ultimately improving surgical decision-making, and patient outcomes. Further validation of CANT1 and B3GNT3 in clinical settings could establish their role as valuable adjuncts in diagnostic pathology and guide more personalized treatment strategies.

This study has several limitations that should be acknowledged. First, the cases were randomly selected from archived samples and may not fully represent the overall population distribution of IDC. Notably, there was a higher proportion of patients younger than 50 years and an absence of grade 1 tumours, which may limit the generalizability of our findings regarding expression frequencies. Additionally, most cases were locally advanced or Stage 3, potentially influencing survival outcomes. Treatment regimens varied among patients, but detailed therapy data were unavailable, which may have confounded survival analyses. Furthermore, PFS was used as the primary survival endpoint, as progression data were more reliably available for this retrospective cohort. However, DFS could not be assessed due to limited data on recurrence in early-stage cases. Although our study highlights the association of CANT1 and B3GNT3 expression with poor prognosis and LNM in IDC, their potential as therapeutic targets requires further validation. Functional studies and clinical trials are necessary to confirm their role in treatment strategies. Taken together, these limitations indicate that this study should be considered exploratory.

### Future directions

Further studies with larger cohorts and functional analyses are needed to validate the precise role of CANT1 and B3GNT3 in IDC and to determine whether they can serve as predictive tools for personalized surgical and therapeutic approaches. A deeper understanding of their biological roles may reveal novel opportunities for targeted interventions in IDC. Additionally, assessing DFS is particularly valuable in early-stage IDC, as it offers important insights into recurrence risk within a relatively short follow-up period. Such studies will help clarify the prognostic value of CANT1 and B3GNT3 expression in different clinical contexts and stages of IDC. Integrating these biomarkers with established clinicopathological factors may further enhance personalized treatment strategies and improve patient outcomes.

## Data Availability

All data analysed during this study are included in this article.

## Abbreviations

LNM: Lymph node metastasis.
CANT1: Calcium-activated nucleotidase 1.
B3GNT3: Beta-1,3-N-acetylglucosaminyltransferase 3.
IDC: Invasive ductal carcinoma.
PFS: Progression free survival.
ECM: Extracellular matrix.
AJCC: American Joint Committee on Cancer.
NPI: Nottingham prognostic index.
TILs: Tumour-infiltrating lymphocytes.
DCIS: Ductal carcinoma in situ.
LVI: Lymphovascular invasion.
LNR: Lymph node ratio.
ER: Estrogen receptor.
PR: Progesterone receptor.
PBS: Phosphate-buffered saline.
DAB: 3,3-Diaminobenzidinetetra hydrochloride
SD: Standard deviation.
HCC: Hepatocellular carcinoma
